# Semiconducting Behaviour and Corrosion Resistance of Passive Film on Corrosion-Resistant Steel Rebars

**DOI:** 10.3390/ma15217644

**Published:** 2022-10-31

**Authors:** Shanglin Lv, Kefei Li

**Affiliations:** 1 Department of Civil Engineering, Tsinghua University, Beijing 100084, China; 2 National Construction Steel Quality Test and Inspection Centre, Inspection and Certification Co., Ltd. MCC, Beijing 100088, China; 3 Central Research Institute of Building and Construction, China Metallurgical Group Cooperation Co., Ltd., Beijing 100088, China

**Keywords:** corrosion-resistant rebar, passive film, semiconducting behaviour, chloride threshold concentration

## Abstract

Chloride-induced corrosion of steel rebars is one of the major causes of the premature failures of reinforced concrete structures served in different environments. This paper investigates the semiconducting behaviour and corrosion resistance of the passive film formed on the corrosion-resistant rebars exposed to simulated concrete pore solutions with different pH values and chloride concentrations. The electronic properties of the passive film were studied using potentiodynamic measurements and capacitance measurements (Mott–Schottky analysis). The results indicate that, firstly, the passive film of corrosion-resistant steel rebar shows n-type semiconducting behaviour with shallow and deep donor states in the band gap during passivation, and the deep donor energy level of corrosion-resistant steel rebar passive film is not sensitive to the decreasing pH value; secondly, under the same conditions, the passive film of corrosion-resistant rebars has a larger negative flat-band potential and thicker space charge layer than hot-ribbed rebars; thirdly, n-type semi-conductivity at a higher potential disappears once the chloride concentration at the rebar surface attains the chloride threshold value; and finally, a reverse charge layer forms on the surface of CR rebar at 0.50 V potential.

## 1. Introduction

A passive film forms on the surface of steel rebars when steel rebars are exposed to an alkaline environment in hardened concrete, protecting the steel from corrosion. Chlorides in the external environment can penetrate the concrete, destroy the passive film, and induce pitting corrosion when the chloride concentration on the rebar surface accumulates to a threshold value [1,2,3]. The corrosion resistance of steel bars can be effectively improved by alloying other elements in the base metal [4,5,6], forming high-performance steel of different types, such as stainless steel [7], weathering steel [8], and corrosion-resistant steel [9]. In civil engineering, stainless steel (SL) rebars have been applied in concrete structures exposed to highly corrosive environments [10]. Weathering steel is mainly used in steel structures and relies on the dense rust layer for corrosion protection. The contents of the corrosion-resistant (CR) rebars alloy elements Cu, P, Cr, and Ni are lower than SL rebars, i.e., 1.0–7.0%, and expect to provide higher corrosion resistance than plain steel rebars [9]. The corrosion resistance of steel rebars in concrete is closely related to the passivation-depassivation process on the rebar surface [11]. Accordingly, the properties of the passive films on the CR steel rebars are crucial for understanding their corrosion behaviour and predicting the service life of engineering structures using these rebars.

So far, the composition and structure of the passive film have been investigated through electrochemical techniques for steel rebars with different surface states. The thickness of the passive film formed on carbon steel rebars was measured as between 5 and 13 nm. The inner part of the film near the substrate mostly contains Fe^3+^ oxides and the outer part of the film contains mainly Fe^2+^ oxides [12]. The steel surface films with mill scales were found to contain mainly the hematite (α-Fe_2_O_3_), the maghemite (γ-Fe_2_O_3_), and the magnetite (Fe_3_O_4_) after exposure to a synthetic pore solution for 2 months [13], and the composition was further identified as α-FeOOH, β-FeOOH, γ-FeOOH, and γ-Fe_2_O_3_ after exposure to simulated concrete pore solution for 144 h [14]. The amount of FeOOH in the outer layer of passive film on carbon steel was found to increase with the addition of chloride ions in different solutions [15]. The passive films on SL steel were found to consist of an iron-enriched outer hydrated layer, and an enriched inner anhydrous layer of chromium oxide and the Cr content in the inner layer increased as the pH value decreased [16]. Although extensive studies have been made on the electronic properties of several kinds of steel in different conditions, they cannot be used to represent the passive film of corrosion-resistant steel rebar. For example, the electronic properties of the passive film are further investigated [17,18,19], and the Mott–Schottky (M-S) theory is employed to analyse the semiconducting behaviour of passive film [20,21]. The passive films of carbon steel rebars all showed n-type semiconducting behaviour with two discrete donor species in simulated concrete pore solutions [22,23], not significantly affected by the addition of sulphates. However, the effect of the low pH value on the performance of the passive film is not considered in solutions with different chloride concentrations, and it is necessary to further confirm these conclusions with extended pH. The passive film of weathering steel in a slightly alkaline aqueous solution presents n-type semiconducting behaviour in laboratory test solutions [24]. The passive film of 316LN stainless steel demonstrated both p-type and n-type semiconducting behaviour, and the addition of nitrogen modified the oxide layers of the passive film [25]. The capacitance behaviours of austenitic stainless steel (AISI 304) and ferritic stainless steel (AISI 446) had significant differences, and the passive film became very thin and highly doped at low pH values [26,27]. The electronic properties of the passive film of high-chromium CR steel were similar to the stainless steel 316LN, showing both n-type (positive slope) and p-type (negative slope) semi-conductivity [28]. However, quantitatively expressing the corrosion extent by studying the electronic properties is not carried out.

Nevertheless, the properties of the passive film formed on CR rebars have not yet been studied systematically, especially the semi-conducting behaviours. Motivated by this need, this paper investigates the semi-conducting behaviours of passive film on CR steel rebar under different combined conditions of pH values and chloride concentrations. Accordingly, this paper is organized as follows: the materials, specimens, and experimental procedures are presented in Section 2; the impact of pH values on passive film is studied via the M–S theory in Section 3; Section 4 is dedicated to the impact of chlorides on passive film through the threshold concentration of chlorides at depassivation; further analyses are given in Section 5; and the conclusions are provided in Section 6.

## 2. Materials and Methods

### 2.1. Materials and Specimens

Four kinds of steel rebars with different chemical compositions and production processes are studied in this work. They are corrosion-resistant (CR) steel rebar (HRB400c) [9], hot-rolled (HR) steel rebar (HRB400) [29], quenching and self-tempering (QT) ribbed rebar (RRB400) [30], and stainless-steel (SL) rebar (HRB400s) [31], respectively. The rebars with a diameter of 16 mm were cut into 10 mm thick pieces for electrochemical test specimens. After being cut to size, the specimens were cleaned with alcohol to remove rust and grease and were then rinsed with deionised water. The cross sections of the specimens were ground to 2000# with sandpaper, followed by a polish to 2 μm using a polish machine to eliminate the heterogeneities of the surface. After the polishing process, the specimens were stored in a desiccator to avoid corrosion before the test. The chemical compositions of the specimens are listed in Table 1.

### 2.2. Simulated Pore Solutions

In practical engineering, the concrete pore solution is a mixed electrolyte solution that is composed of saturated Ca(OH)_2_ and multiple aqueous ion species such as Na^+^, K^+^, and OH^−^ [32]. The focus of this paper is to study the semiconductor performance and corrosion resistance of the passive film formed on the CR rebar under conditions of different pH values and chloride concentrations. The present study does not use the real concrete pore solution, but rather instead saturated Ca(OH)_2_ to reduce the influence of other ions so that the effect of pH and chloride on the semiconducting behaviour and corrosion resistance of passive film on CR rebar can be studied in a systematic and controlled way [22]. The NaHCO_3_ was added to the saturated Ca(OH)_2_ solution to adjust pH values (8.5, 10.5, and 12.5) for the simulated solutions. During the preparation, the solution container was sealed to prevent the carbonation from CO_2_ in the air, and a magnetic stirrer was used to ensure the homogeneity of the liquid solution. The pH value of the solution was checked with a pH meter before and after each test to verify that the solution remained chemically stable.

**Figure 1:**
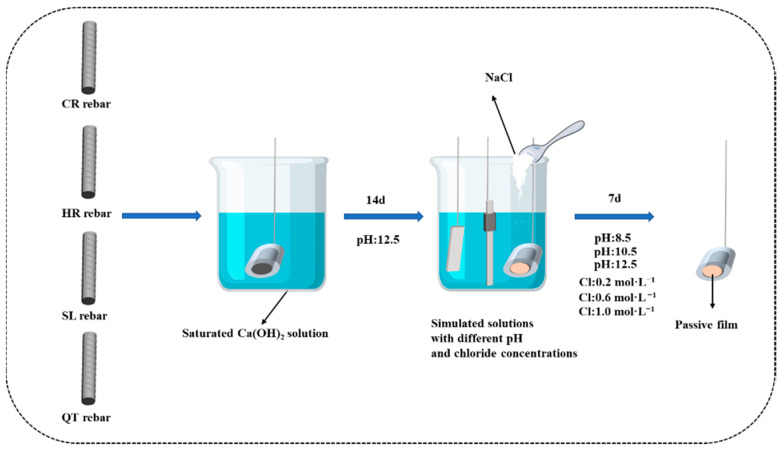
Schematic diagram of the preparation process of passive film in simulated solutions.

The specimens of CR, HR, QT, and SL rebars were firstly immersed in the 12.5 pH solution for 14 d to allow the growth of passive films on the surface of rebar specimens. Then, the specimens were immersed into simulated solutions with different pH values (12.5, 10.5, and 8.5) and chloride concentrations (0.2 mol·L^−1^, 0.6 mol·L^−1^, and 1.0 mol·L^−1^) for 7 d to leave the test solution fully equilibrated with the passive film on the rebar specimens, as shown in Figure 1. The electrical and electrochemical properties were measured at the end of this period of immersion.

When testing the chloride threshold concentrations, firstly, the CR, HR and QT specimens were soaked in the simulated solutions with different pH values (8.5, 10.5, and 12.5) for 10 d. Then, the sodium chloride was added to the test solutions, increasing the concentration by 0.02 mol·L^−1^ every 2 d. After that, the corrosion potential was measured after the OCP became stable.

### 2.3. Electrochemical Measurements

The electrochemical measurements were conducted using a Princeton PAR273A workstation (Metek, Oak Ridge, TN, USA) via a three-electrode system containing a working electrode, a platinum auxiliary electrode, and a saturated calomel electrode (SCE) as the reference. The potentials measured hereafter refer to this SCE. The rebar specimen, acting as a working electrode, was immersed in the simulated solution. The lateral surface of the specimen was coated by epoxy resin with an exposed working area of 2 cm^2^. The measurements were conducted once the open circuit potential (OCP) of the working electrode became stable. Three parallel specimens were prepared for each test for good reproducibility, and a group of data was selected to study for clarity.

The semiconductor properties of the passive films were determined by the capacitance measurements after the M–S tests. The M–S tests were carried out at a fixed frequency of 1 kHz to reduce the influence on the space charge capacitance by using a sinusoidal voltage excitation signal with a disturbance amplitude of 5 mV vs. OCP and a scanning rate of 10 mV·s^−1^. The corrosion resistance of the passive film against chlorides was measured by potentiodynamic curves. After the electrode OCP was stabilized, the polarisation curves were measured with the scanning potential in the range of −1.5–1.0 V vs. OCP with the scanning rate of 0.332 mV·s^−1^. The potential was scanned from positive to negative to ensure the correct measurement of the flat band potential. All the electrochemical measurements were repeated three times to check the reproducibility.

## 3. Impact of pH Value on Passive Films

### 3.1. Passivation Behaviour

**Figure 2:**
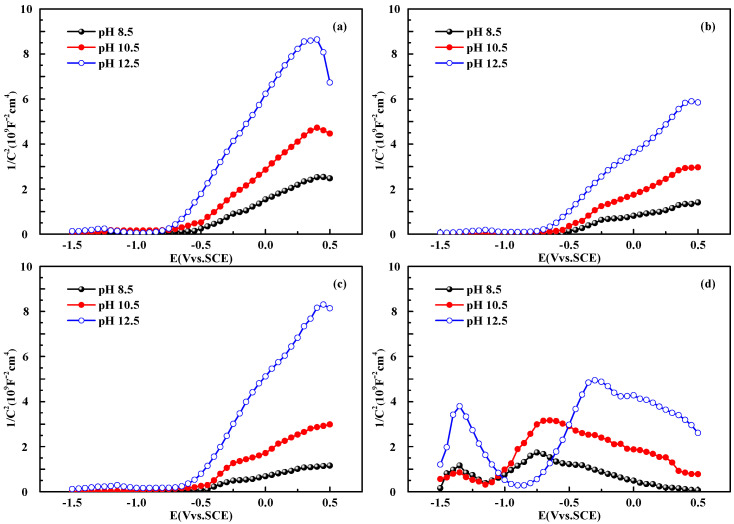
M–S curves of passive films of CR (**a**), HR (**b**), QT (**c**), and SL (**d**) rebars in simulated solutions with different pH values (12.5, 10.5, and 8.5).

Figure 2 provides the M–S curves of the passive films of CR, HR, QT, and SL rebars in simulated solutions at different pH values (12.5, 10.5, and 8.5). The M–S curves of the passive films of the CR, HR, and QT rebars show positive slopes, indicating that the passive films exhibit n-type semiconducting behaviour [33,34]. It also means that the impurity state is mainly shown as the donor state in the passive film of CR, HR, and QT rebars, and the majority of charge carriers in the space charge layer are electrons. After the free electrons of the n-type semiconductivity have consumed, a positively charged depletion layer is formed in the region [35,36]. The M–S plots of the SL rebars in different pH solutions show four linear regions with different slopes, which is related to the chemical compositions of films [37,38]. From the literature [39], the n-type semiconducting behaviour of passive film on SL rebars can be ascribed to the outer layer of the film, which is mainly composed of CrO_3_ (CrO_4_^2−^) and Fe_2_O_3_ compound, while the p-type semiconductor behaviour is induced by an inner layer principally consisting of Cr-oxides or hydroxide. Unlike SL rebars, CR rebars are not observed to demonstrate p-type semiconducting behaviour, indicating that Cr_2_O_3_ does not form in the passive film. Due to the different nature of the passive film semiconductivity of SL rebars, the SL rebar results will not be discussed further, and the properties will only be compared among the CR, HR, and QT specimens.

In Figure 2, the M-S curves of the CR, HR, and QT rebars have an inflection point near −0.25 V and show nonlinear behaviours. In the literature, this nonlinear behaviour is attributed to the second donor state or the surface roughness [40]. Since the surface of rebar specimens has been polished carefully in this study, this nonlinear behaviour is more likely due to the existence of the second donor state in the band gap of the passive film. The ionization of deep donor impurities in passive films was reported to cause an increase in the charge carrier concentration [41], which is consistent with the M–S curves in Figure 2. For the passive film on the steel surface, the shallow and deep donor states are caused by the oxidation of iron ions at the tetrahedral and octahedral positions in the inverse spinel lattice [42,43,44]. The tetrahedral sites correspond to the shallow energy levels occupied mainly by Fe^3+^, and the octahedral sites correspond to the deep energy levels occupied by Fe^2+^ and Fe^3+^ [43]. Due to the lower bond energy at tetrahedral sites, the ionization is more likely to occur at lower applied potentials, resulting in more unstable properties. The capacitance gradually increases with lower pH values, caused by the thinning of the passive film and the subsequent increase in the film’s porosity [41]. Under the same pH value, the passive film of the CR rebars has lower capacitance compared with the HR and QT rebars, indicating that the passive film of CR rebars is denser and thicker.

### 3.2. Donor Density

The capacitive behaviour of the passive film is affected by impurities of both the shallow and the deep donor states. For an n-type semiconductor, the relationship between the slope of the M–S curve and the shallow and deep donor densities can be derived from [45]:

(1) $$\frac{1}{C^{2}}=\frac{1}{C_{H}^{2}}+S_{1,2}\left(E-E_{fb}-\frac{kT}{e}\right)$$

(2) $$N_{D1}=\frac{2}{\varepsilon \varepsilon_{0}eS_{1}}\;N_{D2}=\frac{2}{\varepsilon \varepsilon_{0}eS_{2}}-N_{D1}$$

Here, *C* and *C_H_* are the capacitance and the Helmholtz capacitance of semiconductor electrode (F m^−2^), *E* is the electrode potential (V vs. SCE), *E_fb_* is the flat band potential (V vs. SCE), *ε* is the relative dielectric constant taking 15.6 (CR rebar [44]) and 12.0 (HR and QT rebars [42]), *ε*_0_ is the vacuum dielectric constant (8.85 × 10^−12^ Fm^−1^), *k* is the Boltzmann constant (1.38 × 10^−23^ J K^−1^), *T* is the absolute temperature (K), *e* is the electric charge (1.60 × 10^−19^ C), *N_D_*_1,2_ is the donor density of the shallow and deep donors (cm^−3^), and *S*_1,2_ is the slope of the M-S curve of the shallow and deep donor regions.

**Figure 3:**
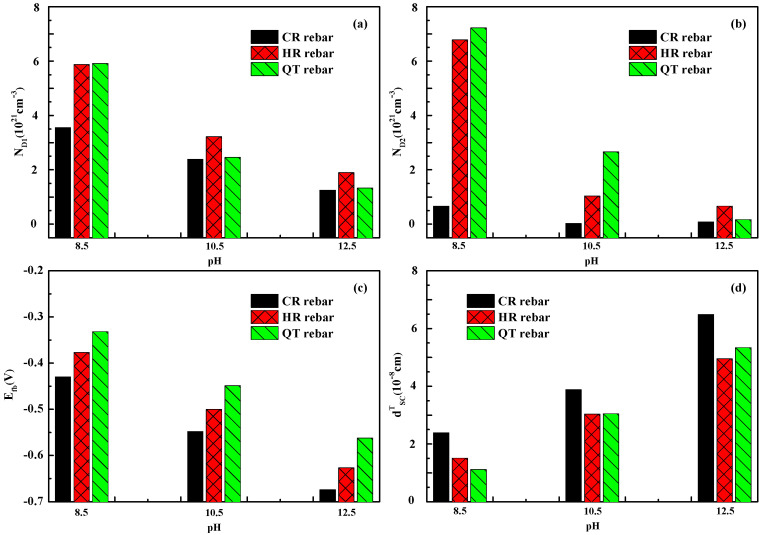
Electronic properties parameters of passive films in simulated solutions of CR, HR, and QT rebars at different pH values (12.5, 10.5, and 8.5): (**a**) $N_{D1}$, (**b**) $N_{D2}$, (**c**) $E_{fb}$, (**d**) $d_{SC}^{T}$.

From Figure 2, the voltage range of the CR, HR, and QT rebars at the shallow donor region is −0.75~−0.25 V and the voltage range at the deep donor region is between −0.25 and –0.5 V in the different pH solutions. The potential of −0.25 V at the inflection point is the transition potential (E_t_) of the shallow and deep donor regions. Figure 3 provides the electronic properties of the passive film of the CR, HR, and QT rebars. The shallow donor density, *N_D_*_1_, of the CR, HR, and QT rebars increase by an order of magnitude when the pH value decreases in the simulated solutions. The deep doner density, *N_D_*_2_, of the HR and QT rebars increase exponentially with the decrease in the pH value, while the *N_D_*_2_ of the CR rebar remains stable with the change of pH value. The *N_D_*_1,2_ values of CR rebars are lower than HR and QT rebars in all cases. The conductivity of the passive film depends on the carrier concentration in the passivation film: the lower the carrier concentration, the more difficult it is for electrons to conduct in the passive film [22,37,43]. Compared with the HR and QT rebars, the passive film resistance of CR rebars is higher due to the lower donor densities, indicating that the CR rebar has stronger resistance to electron conduction, especially under low pH values (8.5).

Compared with *N_D_*_1_, the deep donor densities, *N_D_*_2_, of the HR and QT rebar are more sensitive to the change in pH value. As the pH value decreases from 12.5 to 8.5, the $N_{D2}$ increases by one order of magnitude, indicating that the structure of the passive film is notably altered during the pH value decrease. This alteration is caused due to the formation of unstable Fe_3_O_4_ with the important amount in the passive film of the HR and QT rebars during the pH value decrease. The oxide Fe_3_O_4_ presents an inverse spinel structure, of which one-third are tetrahedral sites occupied by Fe^3+^ and the other two-thirds are octahedral sites occupied by Fe^3+^ and Fe^2+^ [43,45], respectively. The formation of Fe_3_O_4_ causes the octahedral sites in the passive film to increase significantly, so the *N_D_*_2_ values of the HR and QT rebars increase rapidly with the decreasing pH value. However, the *N_D_*_2_ of the CR rebar passive film is not sensitive to the pH value change, and the M–S curves present linearity in a much wider range of voltage. This is due to the presence of alloy elements, such as Cr, Cu, P, and Ni, in the CR steel: the formation of densely packed amorphous ferric oxyhydroxide can be promoted by alloy elements such as Cr, Cu, P, and Ni [46,47,48,49], the elements Cr and Ni will concentrate at the defects of the passive film and grain boundaries [46,47,48], and the Cu and P can promote the formation of α-FeOOH phase in the internal rust layer to inhibit the Fe_3_O_4_ formation [49]. All these factors help to enhance the density and stability of the passive film of the CR rebars.

### 3.3. Flat Band Potential and Space Charge Layer Thickness

The flat band potential, *E_fb_*, of the passive film of rebars refers to the electrode potential when the curvature of the passive film’s interface energy band is zero [37]. When the steel rebar is in contact with the simulated solution, the energy level difference between them causes a transfer of electrons until an equilibrium is reached. Compared with the simulated solution, the charge carrier concentration in the passive film is lower, so the space charge layer and the built-in electric field are formed, leading to the energy band bending of the passive film interface [25]. Under the applied voltage, the electrode potential is the flat band potential of the steel rebar if the Fermi energy level of the electrons in the passivation film is equal to that of the ions in the simulated solution. The properties of the steel passive film and the composition of the simulated solution jointly determine the value of the flat band potential [45]. The charging state of the passive film can be judged by the flat band potential. When the electrode potential is higher than the flat-band potential, *E* > *E_fb_*, a depletion layer is formed at the interface between the passive film and the solution. In this case, the Fermi level is bent downward, and the surface of the passivation film is positively charged. Otherwise, when the electrode potential is lower, *E* < *E_fb_*, an accumulation layer is formed at the interface between the passive film and the solution. In this case, the Fermi level is bent upward, and the surface of the passivation film is negatively charged [50]. For a passive film with high carrier concentration, the flat band potential is determined through Equation (1) by letting 1/C^2^ = 0, as shown below:

(3) $$E_{fb}=E_{0}+\frac{\varepsilon \varepsilon_{0}eN_{D1}}{2C_{H}^{2}}-\frac{kT}{e}$$

where *E*_0_ is the potential value at the intersection of the M–S curve of the shallow donor region and the horizontal axis. To determine the flat band potential value more accurately, Equation (3) considers the effect of Helmholtz capacitance *C_H_* = 20 μF cm^−2^ on the capacitance value [51]. The regressed *E_fb_* values are given in Figure 3. From Figure 3, the flat band potential of CR is more negative than those of the HR and QT. The negative shift of the flat band potential indicates the Fermi level increase of the semiconductor and the increase of the pitting potential [25,52]. Therefore, the electrons in the conduction band are more difficult to lose, resulting in the enhanced corrosion resistance of CR rebars. As the pH value decreases, the flat band potential shifts positively. The pH value of the simulated solution influences the dissociation balance of H^+^ in the passive film, and dipoles are formed at the interface, shifting the water redox potential in the solution. The positive shift potential of the CR rebar was about −0.12 V from pH = 12.5 to 10.5 or pH = 10.5 to 8.5, similar to the result of −59 mV/pH in the literature [50].

Assuming the space charge layer capacitance, *C_SC_*, of the semiconductor conforms to the simple plate capacitor model and ignoring the Helmholtz electric double layer capacitance, one can express the space charge layer thickness, *d_SC_*, through [53]:

(4) $$d_{SC}=\frac{\varepsilon \varepsilon_{0}}{C_{SC}}$$

Using Equation (1), the space charge layer thickness can be written as:

(5) $$d_{SC}={\left[\frac{2\varepsilon \varepsilon_{0}}{eN_{D1}}\left(E-E_{fb}-\frac{kT}{e}\right)\right]}^{1/2}$$

The thicknesses of the space charge layer, *d^T^_SC_*, at the transition potential of the shallow and deep donor regions are calculated and given in Figure 3. The *d^T^_SC_* values of CR, HR, and QT passive films decrease with the decrease of pH values. The thickness *d^T^_SC_* of HR and QT passive films range from 1 Å to 5 Å, which is consistent with the results for carbon steels [22,43]. Under pH = 12.5, the space charge layer thickness of CR reaches 6.39 Å, exceeding the range of carbon steel thickness. This larger thickness also confirms that the passive film of CR rebars has better stability and higher corrosion resistance.

## 4. Impact of Chlorides on Passive Films

### 4.1. Chloride Threshold Concentration and Passivation Behaviour

The chloride threshold concentrations of CR, HR, and QT rebars at different pH values were measured following the standard [54]. The aqueous chlorides are regarded as reaching the depassivation concentration when the corrosion potential drops rapidly, down to the range of −350 mV and −400 mV. The chloride threshold concentration of CR, HR, and QT under different pH values are thus measured and provided in Table 2.

**Figure 4:**
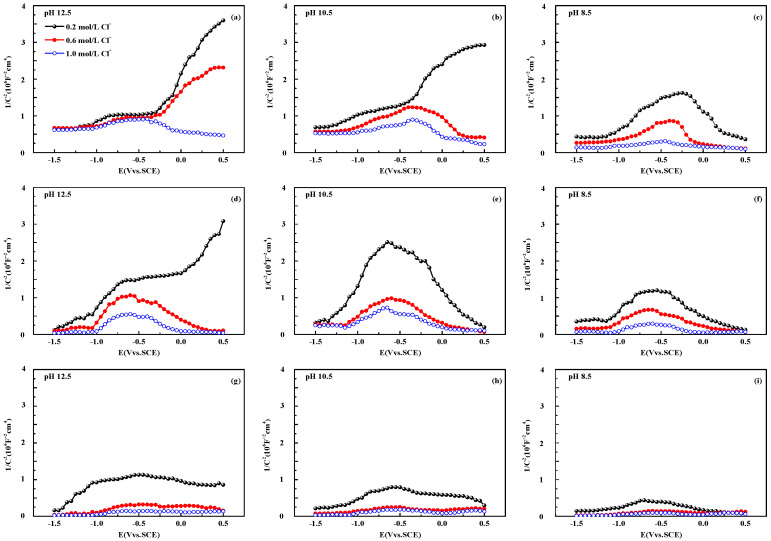
M–S curves of passive films in simulated concrete pore solutions of CR rebars (**a**–**c**), HR rebars (**d**–**f**), and QT rebars (**g**–**i**) at different pH values (12.5, 10.5, and 8.5) and different chloride concentrations (0.2 mol·L^−1^, 0.6 mol·L^−1^, and 1.0 mol·L^−1^).

Figure 4 provides the M–S curves for simulated solutions with different pH values and different chloride concentrations. The addition of chlorides changes the shape and the position of the M–S curves significantly. The curves are no longer linear and the capacitance of the space charge layer increases, indicating that the charge on the surface of the passive film is changed by chlorides [22,43,55]. Following the PDM theory [20,56,57,58], with the addition of chloride ions, many cation interstitials will be formed at the surface of the passive film, increasing the carrier concentration. The passive film under the accumulation of the cation interstitials continuously dissolves and loose pores appear on the surface, which further increases the number of surface defects. This further promotes the adsorption of Cl^−^, increasing the impurity contents in the passive film. The ionization of impurities leads to an increase in capacitance [22,59], which increases the conductivity and deteriorates the corrosion resistance of the passive film. From Figure 4, when pH = 12.5 and the chloride concentration is 0.2 mol·L^−1^ or 0.6 mol·L^−1^, the CR rebar exhibits n-type semiconducting behaviour. When pH = 10.5 and the chloride concentration is 0.2 mol·L^−1^, the CR rebar still exhibits n-type semiconducting behaviour, agreeing with the chloride threshold value in Table 2. In other terms, as the chloride concentration stays within the threshold value, the passive film can maintain a passivation state. For HR and QT rebars, when pH = 12.5 and the chloride concentrations are 0.6 mol·L^−1^ and 1.0 mol·L^−1^, the passive film no longer exhibits n-type semiconducting behaviour at higher potentials. These chloride concentrations have surpassed the threshold values, c.f., Table 2; the cation interstitials accumulate in the passive film, dissolving the oxides and cracking the surface of the passive film [57,58]. At the same time, the composition ratio of α-FeOOH, β-FeOOH, γ-FeOOH, and Fe_3_O_4_ in the passive film changes [60], so the semiconducting properties of the passive film gradually deteriorates and the passive film is altered to an amorphous structure.

Therefore, the M–S curves of CR rebar present n-type semiconducting behaviours at a higher potential, which proves the existence of a stable passive film on the surface of CR rebar. When the chloride concentration exceeds the threshold value, the n-type semiconducting behaviour ceases, and the passive film enters the depassivation state. In summary, the loss of n-type semiconducting behaviour of the passive film of CR rebar requires higher chloride concentrations compared to HR and QT rebars, indicating that the passive film of the CR rebar has better corrosion resistance. The M–S curve characteristics and the critical chloride concentrations are in good agreement. The passive films of HR and QT rebars have similar semiconducting properties and the performance of the HR rebars is slightly better. Therefore, the next section will focus on the corrosion resistances of CR and HR rebars.

### 4.2. Effect of Chlorides on Depassivation

**Figure 5:**
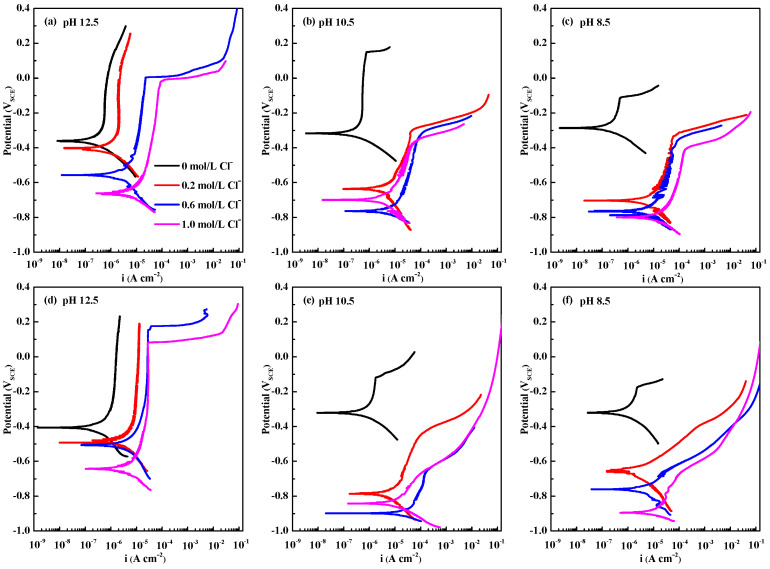
Polarisation curves of passive films in simulated concrete pore solutions of CR rebars (**a**–**c**) and HR rebars (**d**–**f**) at different pH values (12.5, 10.5, and 8.5) and different chloride concentrations (0 mol·L^−1^, 0.2 mol·L^−1^, 0.6 mol·L^−1^, and 1.0 mol·L^−1^).

The polarisation curves of the CR and HR rebars are presented in Figure 5. Table 3 shows the regressed values for the corrosion potential *E*_corr_, corrosion current density *i*_corr_, critical passive current density *i*_cp_, critical passive potential *E*_cp_, and pitting potential *E*_b_ of the CR and HR rebars from these polarisation curves. Low pH values and high chloride concentrations promote the corrosion current density *i*_corr_ and critical passive current density *i*_cp_. Under these conditions, the charge transfer resistance of the passive film decreases, and the corrosion resistance of the passive film deteriorates. In addition, the potential *E*_b_ decreases with pH value decreasing and chloride concentration increasing. When the electrode potential is below the pitting potential, *E* < *E*_b_, the generation number of the cation interstitials at the defects is less than the annihilation number, which is insufficient to form pitting conditions [58]. As the potential continues to rise, the electrode potential reaches the transmission region, *E* ≥ *E*_b_, activating the pitting formed by the cation interstitial aggregation [61,62,63]. Then, the dissolution of the oxides occurs on the surface of the passive film, the valence state of the oxides changes during the corrosion and the semiconducting behaviour of the passive film deteriorates accordingly.

In Figure 5, for the CR rebar in the solution with chloride concentration 0.2 mol·L^−1^, by decreasing the pH value from 12.5 to 8.5, the pitting potential *E*_b_ of the passive film decreases from 253.7 mV to −320.9 mV, and the critical passive current density *i*_cp_ increases from 1.12 × 10^−6^ A cm^−2^ to 8.27 × 10^−6^ A cm^−2^. Under the same conditions, when pH = 12.5, the pitting potential *E*_b_ of the HR rebar is 192.7 mV, and the critical passive current density *i*_cp_ is 4.58 × 10^−6^ A cm^−2^, meaning that the corrosion resistance of the HR passive film is lower than CR rebar. Moreover, when pH = 8.5, the HR rebar does not show passivation in solutions with chloride concentrations of 0.2 mol·L^−1^, 0.6 mol·L^−1^, and 1.0 mol·L^−1^. This demonstrates that the passive film on the HR rebar is seriously damaged under these chloride concentrations. Consistent with the M–S curves, the CR rebar passive film has better stability and corrosion resistance than the HR rebar under low pH values.

## 5. Further Analysis

### 5.1. Energy Band Bending under Applied Potential

**Figure 6:**
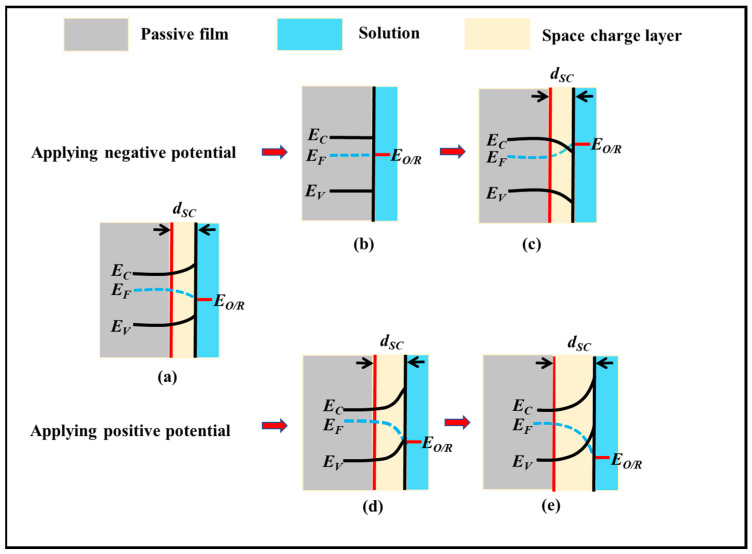
Effect of the applied potential on the band bending: (**a**) the depletion layer state, (**b**) the flat band state, (**c**) the accumulation layer state, (**d**) the space charge layer thickness increases, and (**e**) the reverse charge layer state. *E_F_* is Fermi energy level of passive film, *E_O_*_/*R*_ is the electronic energy level of the redox pair in the simulated solution, *E_C_* is the energy level at the bottom of the conduction band, and *E_V_* is the energy level at the top of the valence band.

This section addresses the mechanisms of the impact of the applied potential on the energy band bending degree and on the semiconductor behaviour of the CR rebar passive film. The applied potential changes the Fermi level of the space charge layer at the interface between the passive film and the simulated solution, which affects the energy band bending at the interface, as shown in Figure 6. The applied potential causes the change of charge carriers, which affects the degree of band bending and the position of the Fermi level relative to the energy band at the interface between the passive film and the solution [45,50,55].

When the CR passive film presents a depletion layer, c.f., Figure 6a, the passive film of the CR rebar demonstrates n-type semiconducting behaviour. When a negative potential is applied, electrons will move into the passive film, causing cathodic polarisation. The addition of electrons will lead to an increase in the Fermi level of the passive film’s space charge layer and a decrease in the space charge layer thickness; at the same time, the curvature of the conduction band and the valence band is reduced. When the applied negative potential reaches the flat-band potential (e.g., −0.67 V at 12.5 pH), the energy band becomes a flat band state, and the thickness of the space charge layer reaches the minimum, c.f., Figure 6b. The passive film is a highly doped, amorphous semiconductor [59]. Compared with the HR and QT rebar, the addition of alloy elements, such as Cr, Cu, P, and Ni, densifies the passive film formed on CR rebars, which reduces the carrier concentration in the passive film [60]. The values in Figure 3 show that the flat band potential of the CR rebar is more negative than those of the HR and QT rebars, indicating that there are fewer crystal defects, such as ion vacancies and substitution ions, in the CR passive film. If the potential remains negative, an accumulation layer is formed as shown in Figure 6c. After that, until the Fermi level of the space charge layer moves into the conduction band, the passive film loses its semiconducting properties and becomes a conductor [50].

When a positive potential is applied to the depletion layer of the passive film of CR rebars, the electrons in the passive film are removed, causing the anodic polarisation. With the positive shift of the potential, the bending degree of the energy band of CR rebar passive film increases, and the space charge layer thickness also increases, c.f., Figure 6d. The passive film demonstrates n-type semiconducting behaviour until about 0.37 V. If the applied potential continues to increase, the passive film will show p-type semiconducting behaviour as shown in Figure 2a. This is because the charge carriers have been exhausted and a reverse charge layer with high positive charge concentration forms on the CR rebar surface as shown in Figure 6e. For the passive film of the HR/QT rebars in simulated solutions at pH = 12.5, the p-type semiconducting behaviour is not obvious even at the applied potential of 0.50 V. Furthermore, the reverse charge layer does not form at pH = 10.5 or 8.5. This could be due to a higher density of the two donor states in the passive film of the HR/QT rebars, which prolongs the depletion of the electron carriers. The space charge layer thickness of the CR rebar is larger than those of the HR/QT rebars, which indicates that both the doping concentration and the conductivity of the passivation film are lower.

**Figure 7:**
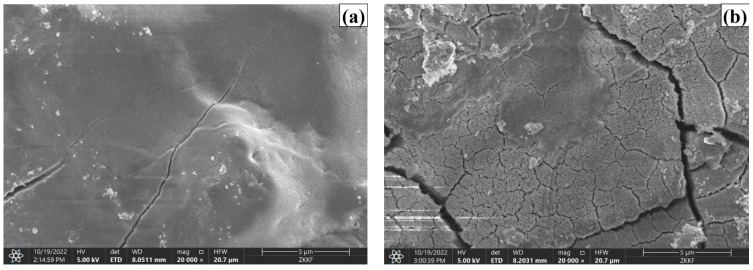
SEM images of the cross sections of the CR and HR rebars exposed to solution with chloride concentrations of 1.0 mol·L^−1^ and pH 12.5: (**a**) CR rebar, (**b**) HR rebar.

The cross sections of the CR and HR rebars exposed to a solution with chloride concentrations of 1.0 mol·L^−1^ and pH 12.5 were observed by scanning electronic microscopy (SEM, JEOL, JSM-7200F). The acceleration voltage was 20 kV, the high vacuum mode was <6 × 10^−4^ Pa, and the resolution was 1.0 nm. As shown in Figure 7a, the CR rebar maintains a more compact passive film in the solution with chloride concentrations of 1.0 mol·L^−1^ and pH 12.5, leading to a low carrier concentration of the passive film. However, Figure 7b demonstrates that a large number of microcracks are distributed on the surface of the HR rebar passive film, resulting in a high doping concentration of the passive film, which leads to higher conductivity.

### 5.2. Pitting Behaviour under Chlorides

**Figure 8:**
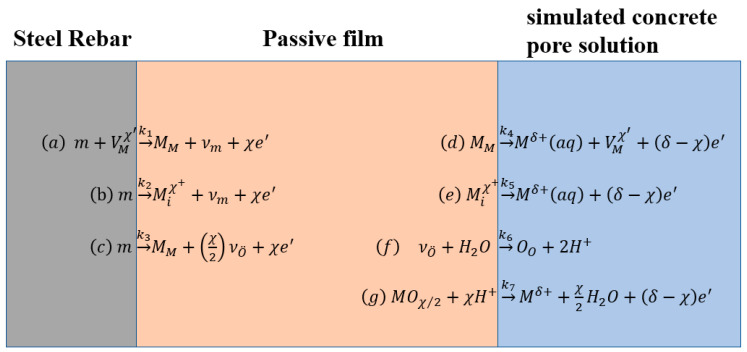
Defect generation and annihilation reactions occurring at interfaces of passive film on CR rebar: *V^χ^*^’^*_M_* is the cation vacancy, *v_m_* is the vacancy in metal substrate, $M_{i}^{\chi^{+}}$ is the cation interstitial, $\nu_{\ddot{O}}$ is the oxygen vacancy, *M^δ^*^+^ (aq) is the cation at passive film/solution interface, *M_M_* is the cation at the cation position on the metal sublattice, *O_o_* is the oxide ion in an anion site on the oxygen sublattice, and MO*_χ_*_/2_ is a stoichiometric passive film oxide.

The pitting behaviour and chloride threshold concentrations of the CR rebar can be analysed through the PDM model [20,56,58,64], presented in Figure 8. The PDM model describes the chloride-induced corrosion with reactions (a)–(g) [20,58,64,65], among which the reactions (c) and (g) describe the generation and dissolution of the passivation film [57].

Similar to HR rebars, the passive film of the CR rebar is a highly doped n-type semiconductor and an oxide with a high degree of point defects, as shown in Figure 3. Its point defects are mainly oxygen vacancies/metal interstitials, i.e., most of *N_D_* and *N_D_*_2_ are metal interstitials. As the pH value decreases, the concentration of metal interstitials gradually increases. In the pitting process, oxygen vacancies are generated at the metal/film interface and consumed at the film/solution interface, while metal interstitials are generated at the film/solution interface and consumed at the metal/film interface. Chloride ions are absorbed into oxygen vacancies at the passive film/solution interface, and cation vacancies are produced by autocatalytic Schottky pair reaction or cation extraction reaction, resulting in an enhanced flux of cation vacancies across the passive film. These two reactions explain why the chlorides need not to be consumed during the chloride-induced corrosion. After the cation vacancies and the matrix iron atoms are met and annihilated, the metal sub-lattice iron atoms pass through the passive film and enter the solution to form iron ions. As shown in Figure 4, the donor density of the CR rebar is lower than that of the HR rebar under the same chloride concentrations, and the loss of n-type semiconducting behaviour of the passive film of CR rebar requires higher chloride concentrations. The above results indicate that the flux of oxygen vacancies and metal interstitials in the passive film of the CR rebar is less than that of the HR rebar. With the increased concentration of chlorides, the cation vacancies accumulate at the steel/passivation film interface when the formation/diffusion number of the cation vacancies is greater than the annihilation number. The accumulation of cation vacancies leads to the formation of blisters at the interface between the passive film and the metal substrate, resulting in the separation of the passive film from the substrate. This prevents the further formation of the passive film so that the passive film around the blister is broken due to dissolution and surrounding stress. The above analysis assumes that only chloride ions enter the oxygen vacancies at the passive film/solution interface, and the formed cation vacancies lead to pitting corrosion. However, if the OH^−^ in the solution enters the vacancy, it will prevent the entry of chloride ions and inhibit the generation of cation vacancies. Therefore, the higher the pH value, the higher the chloride threshold value for the steel rebar.

## 6. Conclusions

According to experimental results and further analysis, the following conclusions can be drawn:

1. The M–S curves of CR, HR and QT rebars demonstrate an n-type semiconducting behaviour with shallow and deep donor states within the band gap during passivation. The deep donor density of CR passive film is less sensitive to the pH value decrease compared to the HR and QT rebars.
2. As the pH value decreases, the passive film of the CR rebar has a more negative flat band potential than those of the HR and QT rebars. The space charge layer thickness reaches 6.39 Å at Et, exceeding the space charge layer thickness range (1 Å to 5 Å) of HR and QT rebars.
3. A higher chloride concentration is required for the CR passive film to lose the n-type semiconductivity, compared to the HR/QT rebars. There is a clear correlation between the M-S curve characteristics and chloride threshold values for different rebars.
4. The CR rebar passive film shows a p-type semiconductor behavior at 0.50 V when the charge carriers have been exhausted and a reverse charge layer is taking form.
5. The CR rebar could be used in the concrete environment where the chloride concentration is less than 0.7 mol·L^−1^ and the pH value is not less than 12.5 to improve the durability of the structure.

## Figures and Tables

**Table 1:** Chemical composition of CR, HR, QT, and SL rebars (wt%).

Specimen	C	Si	Mn	P	S	Cr	Ni	Mo	Cu	N	V
CR rebar	0.090	0.350	0.886	0.026	0.012	1.306	0.748	0.259	0.512	0.0048	0.072
HR rebar	0.228	0.332	1.302	0.0248	0.016	0.031	0.014	0.004	0.040	0.003	0.005
QT rebar	0.186	0.314	1.122	0.0245	0.017	0.210	0.025	0.003	0.020	0.002	0.002
SL rebar	0.012	0.228	0.917	0.003	0.001	24.720	6.120	2.300	0.036	0.160	0.004

**Table 2:** Chloride threshold concentrations for CR, HR, and QT rebars at different pH values.

Rebar	pH Value	Chloride Threshold Value (mol·L^−1^)
CR	12.5	0.72
	10.5	0.26
	8.5	0.16
HR	12.5	0.24
	10.5	0.06
	8.5	0.02
QT	12.5	0.14
	10.5	0.04
	8.5	0.02

**Table 3:** Electrochemical parameters of polarisation curves of the CR and HR rebars at different pH values and chloride concentrations.

Rebar	pH Value	Cl^−^ (mol·L^−1^)	E_corr_/ (mV vs.SCE)	i_corr_/ (A cm^−2^)	i_cp_/ (A cm^−2^)	E_cp_/ (mV vs. SCE)	E_b_/ (mV vs. SCE)
CR	12.5	0	−359.6	1.31 × 10^−7^	2.83 × 10^−7^	−278.2	303.4
		0.2	−398.2	2.73 × 10^−7^	1.12 × 10^−6^	−344.5	253.7
		0.6	−556.0	0.79 × 10^−6^	4.19 × 10^−6^	−467.9	4.0
		1.0	−664.2	2.14 × 10^−6^	0.84 × 10^−5^	−594.6	−20.5
	10.5	0	−316.7	1.43 × 10^−7^	2.64 × 10^−7^	−232.1	159.2
		0.2	−637.4	1.51 × 10^−6^	4.07 × 10^−6^	−591.2	−320.9
		0.6	−698.7	1.32 × 10^−6^	7.82 × 10^−6^	−629.8	−369.2
		1.0	−764.1	2.55 × 10^−6^	1.16 × 10^−5^	−714.6	−417.1
	8.5	0	−286.5	0.96 × 10^−7^	1.98 × 10^−7^	−255.5	−102.1
		0.2	−702.8	2.03 × 10^−6^	8.27 × 10^−6^	−656.7	−334.3
		0.6	−764.1	2.15 × 10^−6^	1.12 × 10^−5^	−729.7	−360.9
		1.0	−799.4	7.21 × 10^−6^	2.05 × 10^−5^	−737,3	−421.4
HR	12.5	0	−409.1	1.35 × 10^−7^	5.78 × 10^−7^	−321.0	228.3
		0.2	−493.8	0.78 × 10^−6^	4.58 × 10^−6^	−409.1	192.7
		0.6	−505.6	1.23 × 10^−6^	1.16 × 10^−5^	−459.5	177.7
		1.0	−644.9	2.33 × 10^−6^	1.28 × 10^−5^	−582.3	86.3
	10.5	0	−321.0	2.14 × 10^−7^	4.33 × 10^−7^	−278.2	−117.8
		0.2	−791.0	3.57 × 10^−6^	8.33 × 10^−6^	−725.6	−417.6
		0.6	−844.7	5.16 × 10^−6^	3.95 × 10^−5^	−872.4	−675.3
		1.0	−891.7	8.63 × 10^−6^	—	—	—
	8.5	0	−324.3	3.31 × 10^−7^	1.04 × 10^−6^	−285.7	−172.1
		0.2	−652.5	1.29 × 10^−6^	—	—	—
		0.6	−756.7	4.33 × 10^−6^	—	—	—
		1.0	−891.7	8.12 × 10^−6^	—	—	—

## Data Availability

Not applicable.
